# Delivering quality health care to people in low-income countries such as Pakistan; the clear link between AMR and UHC

**DOI:** 10.1016/j.amsu.2022.104490

**Published:** 2022-08-25

**Authors:** Muhammad Muzzamil, Mohammad Mehedi Hasan, Maryam Nisa, Shaeroz Raza

**Affiliations:** Health Services Academy, Islamabad, Pakistan; Department of Biochemistry and Molecular 12 Biology, Faculty of Life Science, Mawlana Bhashani Science and Technology University, Tangail, 13 1902, Bangladesh; Health Services Academy, Pakistan; Jinnah Medical and Dental College, Pakistan

Health care is an essential industry that not only highlights national development but is also a necessary component for the long-term growth of our economies and communities. People's well-being improves when they are in good health. Unfortunately, healthcare remains one of Pakistan's most neglected industries, with little progress made in the 75 years since the country's independence. The United Nations Sustainable Development Goals (SDGs) include universal health coverage (UHC) as an aim. This has resulted in UHC being accepted as a national aim by numerous nations in recent years to offer equitably accessible health care [1].

A health card for the whole population of Punjab, Pakistan's most populous province, was announced in January 2022 following a successful trial in Khyber Pakhtunkhwa province. The health card entitles every family to yearly care for around 1 million Pakistani rupees ($5650) in public and private facilities alike [2]. A diagnosis of any communicable or non-communicable disease in Pakistan is closely related to receiving the death penalty due to the significant out-of-pocket expenditures (OOPS) connected with its treatments.

When bacteria can withstand the effects of medications that would typically kill them, they are referred to as drug-resistant or antimicrobial resistant (AMR). AMR causes a decline in the efficacy of antimicrobials (AMs) against the bacteria that are the cause of the infection, results in additional health care expenses, and in the end causes therapy to fail. Antimicrobial resistance may be maintained if the primary health care system is well-functioning. Since people want and require high-quality medical treatment, AMR directly impacts a broken healthcare system. In addition to increasing the number of people affected, this malfunctioning health care system will also raise the cost of UHC. Due to AMR, the number of people affected not only increases, but the burden on the healthcare system will grow as well. Both communicable and non-communicable diseases are already taking a heavy toll on Pakistan, which is currently struggling with the consequences of both diseases [3].

Dr Arif Alvi, Pakistan's president, has called for sweeping reforms to the way antibiotics are administered to people, animals, and the environment, claiming that the overuse of antibiotics is a severe threat to the health of the whole population. According to data from the Global Antibiotic Resistance Partnership, there are too many registered antimicrobial products in Pakistan. Self-medication is common, and doctors prescribe too many antibiotics, which all contribute to drug resistance. Up to ten million people might die each year as a result of medication resistance, and the economic toll could reach £85 trillion by 2050 [4].

More than three medications are prescribed to each patient [5]. General Practitioners (GPs) and public sector institutions with a preference for expensive broad-spectrum antibiotics are more likely to use this inappropriately and indiscriminately. Antibiotics available over the counter (OTC) without a prescription are a frequent practice throughout the nation, leading to a rise in antimicrobial resistance (AMR).

Several investigations in Pakistan have shown evidence of resistance in gram-negative organisms. A third-generation Cephalosporin-resistant Enterobacteriaceae has also been described. Typhoid continues to be a major public health problem across the country because of antibiotic resistance and treatment failure. *Staphylococcus aureus* multi-resistant strains have also been shown to be prevalent in studies throughout Pakistan. Similarly, MDR TB and chloroquine-resistant falciparum are major roadblocks to the respective national and provincial programs’ goals and have serious consequences for the general public [6].

It's already difficult for the post-covid nation to get back on its feet. In addition to affecting the country's future, the additional costs of AMR will have a direct influence on the country's economy and future decisions on health outcomes.

So the government should also create or encourage private health facility investments to guarantee healthcare access. Also, improved teaching platforms for clinicians and patients to learn about antimicrobial resistance and when and how to use antimicrobials appropriately should be made available. Over-the-counter prescriptions should be strictly enforced to ensure that only those who need them may purchase them. When it comes to health care, UHC and AMR are inseparable since they are mutually reliant and essential to the proper functioning of a healthy system.

## Ethical approval

N/A.

## Sources of funding for your research

None.

## Author contribution

Muhammad Muzzamil: Conceptualization, Writing – original draft. Mohammad Mehedi Hasan: Writing - review & editing. Maryam Nisa and Shaeroz Raza: Writing and editing the final draft.

## Registration of research studies

1. Name of the registry: N/A
2. Unique Identifying number or registration ID: N/A
3. Hyperlink to your specific registration (must be publicly accessible and will be checked): N/A

## Guarantor

N/A.

## Consent

N/A.

## Declaration of competing interest

None declared.
